# Biogenic Amines and Food Quality: Emerging Challenges and Public Health Concerns

**DOI:** 10.3390/foods9070859

**Published:** 2020-07-01

**Authors:** Giulia Tabanelli

**Affiliations:** Department of Agricultural and Food Sciences, University of Bologna, 40127 Bologna, Italy; giulia.tabanelli2@unibo.it; Tel.: +39-347-032-8294

**Keywords:** biogenic amines, histamine food poisoning, food quality and safety, decarboxylase bacteria

## Abstract

In addition to pathogenic bacteria and viruses, some bioactive compounds and natural toxins such as biogenic amines (BAs) can be responsible for food poisoning. These compounds, produced mainly by bacteria through the action of decarboxylases, represent a risk for consumers’ health and are involved in several pathogenic syndromes, with histamine and tyramine being the most dangerous ones. Since the presence of dangerous amounts of BAs is associated with the relevant growth of spoiling decarboxylating microorganisms, BA content has been proposed as a food quality index in fresh products. Several factors, both intrinsic and technological, can regulate BA accumulation in foods influencing the decarboxylase-positive bacteria population and proteolysis phenomena, especially in fermented products where strains belonging to different species and genera, commonly found in these foods, have been characterized for their decarboxylase activities and have been associated with high levels of BAs. Due to their impact on human health and food quality, both the development of simple and rapid methods for BA detection and the increase of knowledge of factors involved in BA accumulation are needed to face new challenges in food chains and to reduce health concerns regarding food poisoning.

One of the major challenges in modern times is to produce adequate amounts of safe food, not containing harmful residues, pesticides, and allergens and not contaminated by pathogens (bacteria, virus or protozoa). In this perspective, it is crucial to ensure that the consumed food is not contaminated with potentially harmful elements at any point along the food chain during production, distribution, preparation, and consumption. Despite considerable progress in increasing food safety in recent years, there are still considerably high numbers of food illnesses; it is estimated that almost 1 in 10 people in the world fall ill after eating contaminated food [1,2]. These numbers are even likely underestimated, because of the difficulty in establishing a causal relationship between food contamination and illnesses [3,4].

In addition to pathogenic bacteria and viruses, some bioactive compounds and natural toxins can be responsible for food poisoning. Among these, biogenic amines (BAs) such as histamine, tyramine, cadaverine, putrescine, spermidine, or spermine are involved in several pathogenic syndromes, representing a risk for consumer health since these compounds can cause headache, heart palpitations, vomiting, diarrhea, allergy, and hypertensive crises [5,6]. BAs are organic bases produced mainly by bacteria through the action of decarboxylases, which act selectively on amino acids by removing the carboxyl group with the formation of the correspondent amine and CO_2_. These substances have been placed under attention by European Food Safety Authority (EFSA), which conducted a qualitative risk assessment concerning their presence in fermented foods in the European Union, indicating concentrations that could induce adverse effects in consumers [7]. However, these toxic effects depend on several factors, such as individual sensitivity, the type of BAs, and the consumption of ethanol or monoaminooxidase inhibitory substances, which interact with aminooxidase enzymatic systems able to detoxify food exogenous BAs [8].

Histamine intoxication is the most toxic and frequently observed effect, producing effects on cardiovascular, gastrointestinal, and respiratory systems (low blood pressure, skin irritation, headache, heart palpitations, asthma attacks, etc.). This symptomatology is known as “scombroid fish poisoning”, a global food safety concern due to consumption of fish of the *Scombridae* and *Scomberesocidae* families (tuna, mackerel, bonito, bluefish, etc.) containing high levels of histamine [6,9]. It is interesting to note that also putrescine and cadaverine are associated with this illness, enhancing the toxicity of histamine [8].

Due to the severity of its symptoms, tyramine is another BA related to food poisoning, causing the so called “cheese reaction”. In fact, this BA is the most frequently found in cheeses, and it is responsible for nausea, vomiting, dietary-induced migraine, increased cardiac output, respiratory disorders, and elevated blood glucose [10,11]. On the other hand, tyramine can be present at high concentrations in meat and meat products, due to the metabolism of tiraminogenic lactic acid bacteria (LAB), which are considered the most efficient producers of tyramine [12,13,14,15].

Since the presence of dangerous amounts of BAs is associated with a relevant growth of spoiling decarboxylating microorganisms (i.e., enterobacteria and pseudomonads), BA content has been proposed by several authors as a food quality index, being an indirect indicator of excessive microbial proliferation, nonfermented food, poor hygienic quality, and scarce food freshness [8]. In particular, different BA-based quality indices have been developed for fish products and meat [16,17], but they are considered less satisfactory for fermented products where BA content varies considerably according to different factors linked to raw material quality, fermentation and ripening processes, use of starter cultures, and other technological factors [18]. All of these conditions directly or indirectly affect decarboxylase positive bacteria population and proteolysis, enhancing substrate availability (free aminoacids), acting with combined effects, and modulating BA accumulation in food.

Although knowledge concerning the factors affecting BA production in fermented foods is well documented and BA content in these products is of great interest not only for its potential health concerns but also from an economic point of view, it is difficult to prevent their accumulation since their production conditions cannot be easily modified. Moreover, strains belonging to different lactic acid bacteria (LAB) species and genera, commonly found in fermented foods (i.e., enterococci, streptococci, and leuconostocs), have been characterized for their decarboxylase activities and have been associated with high levels of these compounds in food [19,20,21,22]. The presence of the decarboxylase genes involved in the production of BAs is mostly strain-dependent rather than species-specific, and, recently, the genes belonging to BA biosynthetic pathways in LAB have been identified and the genetic organization of decarboxylase clusters has been reviewed [11,23,24].

In fermented foods, LAB naturally occurring in spontaneous or natural fermentations and noncontrolled autochthonous nonstarter LAB (NSLAB), consisting mainly of mesophilic facultative or obligate heterofermentative bacteria, which exert a crucial role in maturation phenomena and development of flavor, are usually characterized by a high metabolic heterogeneity and can strongly contribute to BA accumulation [25,26]. For these reasons, the presence of small BA concentrations in fermented foods is unavoidable. Concentrations below 20 mg/kg for alcoholic and nonalcoholic beverages, fermented vegetables and soy products, and up to several hundred mg/kg for some sausages and cheeses, have been reported [7].

Due to their impact on human health and food quality, it is of great importance to prevent the excessive accumulation of BAs in foods and to develop a simple and rapid method for their detection. Numerous analytical methods for BA determination have been studied, i.e., colorimetric and fluorometric methods focused mainly on determining histamine individually, fast commercial kits based on the Elisa enzyme immunoassay (especially for histamine detection in fish), and chromatography methods for the simultaneous determination of several BAs [8]. Among these latter, high-pressure liquid chromatography (HPLC) is the most frequently applied for its high resolution, sensitivity, and versatility, and because it is the specific method reported by EFSA [7].

Despite their strong influence on food quality, there is no specific regulation regarding BA food content, with the exception of histamine in fishery products. European Commission Regulations (2073/2005, 144/2007, and 365/2010) regard some fish species (*Scombridae*, *Clupeidae*, *Eugraulidae*, *Coryphenidae*, *Pomatomidae*, and *Scomberesocidae*) with limits between 100 and 200 mg/kg of histamine for two of nine samples, while Food and Drug Administration sets more restrictive limits [27]. In general, the same limits are suggested also for meat, dairy, or other products in the absence of specific legislation [7,16].

In the last years, an increased scientific effort on biogenic amine studies allowed for the obtaining of deeper knowledge about the genetic and biochemical mechanisms of BA production, highlighting important information about the possibility to reduce the risks associated with their accumulation in food products, both fresh and fermented. Nevertheless, the increasing global food demand requires one to face new challenges in food chains and to reduce health concerns regarding food poisoning. All the actors of food production (production sectors, commercial processors, public institutions, and consumers) must be involved in guaranteeing the quality and safety of food, including the reduction of BA content. From this perspective, increasing knowledge of factors involved in BA accumulation and their combined effects and the use of defined starters or selected autochthonous strain mixtures for fermented products, chosen on the basis of the absence of decarboxylase activity and endowed with peculiar metabolic and functional features such as bioprotection or BA degradation properties, can represent a strategy to prevent the presence and growth of aminobiogenic microorganisms.

The present manuscript is an editorial for a Special Issue book edition, i.e., “Biogenic Amines on Food Safety”, edited by Dr. Claudia Ruiz-Capillas and Dr. Ana Herrero Herranz. This Special Issue contains eleven high-quality papers concerning BA presence in foods, their impact on food safety and quality, and an update of the knowledge on BA metabolisms in bacteria.
